# Obituary: Mark Thomas Fisher, Ph.D. June 21,1954–September 4, 2018

**DOI:** 10.3389/fmolb.2018.00102

**Published:** 2018-12-14

**Authors:** Joseph D. Fontes

**Affiliations:** Department of Biochemistry and Molecular Biology, University of Kansas Medical Center, Kansas City, KS, United States

**Keywords:** obituary announcements, GroE chaperonins, mark fisher, obituaries, obituary

The ticking of a bicycle derailleur in the halls of the department heralded the arrival of Dr. Mark Fisher each morning. With a commitment rivaling the Post Office, his daily commute, often through rain, sleet and snow, was always on two wheels, powered by Mark himself. This daily routine revealed something about Mark, a commitment to what he believed was best for himself and the planet, and a determination to push through any obstacle. As a scientist, family man, colleague and friend, Mark gave everything he had, his passion and warmth filling any room he was in. Mark's life was large and the impacts he made were also large and touched countless people. His passing has left an absence that is just as large.

Mark Thomas Fisher, Ph.D., earned bachelor's degrees in Microbiology and in Chemistry from Purdue University. He was awarded a Ph.D. in Biochemistry under the mentorship of Dr. Stephen G. Sligar at the University of Illinois, Urbana-Champagne, in 1987. His dissertation work involved cytochrome P450 with a focus on characterizing ferric spin equilibria during substrate binding. His graduate training was bookended by the wedding to his wife Kathleen and, just before his graduation, the arrival of his first daughter, Maggie.

Mark moved from Illinois to a post-doctoral fellowship at the NIH in the laboratory of Dr. Earl Stadtman, undertaking studies on the structure of glutamine synthetase (GS) from E. coli. It was during that fellowship that Dr. Fisher first worked with the enzyme that would dominate his career, the protein chaperonin GroEL. He examined the assembly of dodecameric GS in the absence and presence of GroEL. It was also during that time that his family welcomed a second child, William, which Mark argued was the highlight of his post-doc.

Following his time at the NIH, Mark obtained a faculty appointment in the Department of Biochemistry and Molecular Biology in the School of Medicine at the University of Kansas, in 1992. It was during the first hectic years as an assistant professor that his final child, Mallory, was born. He would joke that he stayed in Kansas for the rest of his career because every time he and Kathy moved, they ended up with another child and they decided three was enough. At the time of Mark's passing, he was a Professor in the department.

With GroEL at the center of his research program, Mark's creativity and curiosity found full flower. In particular, his laboratory used GroEL as a tool for capturing transient protein folding intermediates, providing the very first images of a partially folded protein bound to a chaperone protein in the early 2000s. Notably, Mark made early and significant contributions to the notion of kinetic partitioning as the underlying mechanism of GroEL. Mark also made an early contribution to the debate over whether GroELS functions as an active folding agent or simply a passive, aggregation-inhibiting Anfinsen cage, by demonstrating that the aggregation propensity of GS folding intermediates does not reliably predict their folding dependence on GroELS. Mark leveraged GroEL for a range of discoveries including capturing the anthrax toxin pore structure which allowed the first EM imaging of this important structure. In addition, his lab developed a chaperonin-based biolayer interferometry biosensor to detect pre-aggregate species of concentrated protein therapeutics. He later used this same system to validate the efficacy of potential drugs to reverse protein folding diseases.

In addition to his chaperonin based research, Mark's lab developed methods to produce pure membrane proteins solubilized in lipid nanodiscs using the anthrax toxin pore as a primary test protein. As a result of this unique approach, Mark's laboratory was the first in the world to image a membrane protein solubilized in a lipid nanodisc using high resolution EM methods. More recently, his lab succeeded in imaging the first structures of the tetanus toxin bound to lipid nanodiscs using negative-stain electron microscopy.

Mark's lab also developed a unique technical approach to convert bacterial protein toxins to their membrane insertion competent states under endosomal pH conditions, while avoiding aggregation. This ability led to a unique biolayer interferometry platform that allowed monitoring of real time transition kinetics. Mark's work over the years led to over fifty published manuscripts, two patents, countless presentations and invited seminars and the founding of a company, CHAPR_x_ LLC (Chaperone Assisted Protein Rescue_x_). He was a prodigious and generous collaborator, with deep relationships with colleagues a multiple institutions.

Mark was a cherished colleague, beloved for his scientific acumen as much as for his personal warmth and sense of humor. He embodied the role of *Professor*: he sought to understand the natural world and his love for this endeavor was expressed by enthusiastically sharing his knowledge with those around him, whether trainee or colleague. He was committed to the educational programs and research enterprise of the University of Kansas School of Medicine, and spoke with great conviction in public and in committees to defend and nurture those missions. In his professional life, Mark's first commitment was always to his students, and he reveled in his interactions with trainees. His great love for science animated him and he safeguarded this treasure by training generations of scientists and physicians, sharing with them his passion, knowledge, and insights. His professional legacy will persist in his important body of work, the many trainees whose lives he touched, and the colleagues who were better scientists and people for knowing him. His departmental colleagues will truly miss seeing Mark in his riding clothes and helmet, rolling his bicycle through the department halls—perhaps sunburnt, drenched by rain, frozen by snow—but always ready with a hearty greeting and a warm smile. His passions animated him, and overtook him for a brief moment at the end of his life, but the more than six decades that preceded are what truly defined this remarkable man, not his final day. Mark is survived by his wife Kathleen, his three children and two grandchildren. He was a friend to all who knew him and the great sadness born of his absence is a testament to the life he lived so very well.

## Author Contributions

The author confirms being the sole contributor of this work and has approved it for publication.

### Conflict of Interest Statement

The author declares that the research was conducted in the absence of any commercial or financial relationships that could be construed as a potential conflict of interest.

